# Ferulic Acid Supplementation Improves Lipid Profiles, Oxidative Stress, and Inflammatory Status in Hyperlipidemic Subjects: A Randomized, Double-Blind, Placebo-Controlled Clinical Trial

**DOI:** 10.3390/nu10060713

**Published:** 2018-06-02

**Authors:** Akkarach Bumrungpert, Supathra Lilitchan, Siriporn Tuntipopipat, Nednapis Tirawanchai, Surat Komindr

**Affiliations:** 1 Department of Nutrition, Faculty of Public Health, Mahidol University, Bangkok 10400, Thailand; supathra.lil@mahidol.ac.th; 2 Institute of Nutrition, Mahidol University, Nakhonpathom 73170, Thailand; siriporn.tun@mahidol.ac.th; 3 Department of Biochemistry, Faculty of Medicine Siriraj Hospital, Mahidol University, Bangkok 10700, Thailand; nednapis.tir@mahidol.ac.th; 4 Department of Medicine, Faculty of Medicine Ramathibodi Hospital, Mahidol University, Bangkok 10400, Thailand; surat_komindr@yahoo.com

**Keywords:** anti-inflammation, anti-oxidant, ferulic acid, lipid profiles, phenolic compound

## Abstract

Ferulic acid is the most abundant phenolic compound found in vegetables and cereal grains. In vitro and animal studies have shown ferulic acid has anti-hyperlipidemic, anti-oxidative, and anti-inflammatory effects. The objective of this study is to investigate the effects of ferulic acid supplementation on lipid profiles, oxidative stress, and inflammatory status in hyperlipidemia. The study design is a randomized, double-blind, placebo-controlled trial. Subjects with hyperlipidemia were randomly divided into two groups. The treatment group (*n* = 24) was given ferulic acid (1000 mg daily) and the control group (*n* = 24) was provided with a placebo for six weeks. Lipid profiles, biomarkers of oxidative stress and inflammation were assessed before and after the intervention. Ferulic acid supplementation demonstrated a statistically significant decrease in total cholesterol (8.1%; *p* = 0.001), LDL-C (9.3%; *p* < 0.001), triglyceride (12.1%; *p* = 0.049), and increased HDL-C (4.3%; *p* = 0.045) compared with the placebo. Ferulic acid also significantly decreased the oxidative stress biomarker, MDA (24.5%; *p* < 0.001). Moreover, oxidized LDL-C was significantly decreased in the ferulic acid group (7.1%; *p* = 0.002) compared with the placebo group. In addition, ferulic acid supplementation demonstrated a statistically significant reduction in the inflammatory markers hs-CRP (32.66%; *p* < 0.001) and TNF-α (13.06%; *p* < 0.001). These data indicate ferulic acid supplementation can improve lipid profiles and oxidative stress, oxidized LDL-C, and inflammation in hyperlipidemic subjects. Therefore, ferulic acid has the potential to reduce cardiovascular disease risk factors.

## 1. Introduction

Cardiovascular disease (CVD) is an important non-communicable disease and a leading cause of death in South East Asia [1,2]. The major factors that contribute to CVD are an imbalance between free radical production and antioxidant activity [3], LDL-C oxidation [4], and inflammatory status [5], particularly in hyperlipidemic patients [6]. In view of the medical expenditure, CVD treatment is relatively high and tends to increase year by year [7,8]. To lessen the risk of CVD, smoking cessation, regular physical activity, and dietary modification, such as increasing the portion of fruits and vegetables, are recommended. Fruits and vegetables are rich in phytochemicals, which may decrease the CVD risk factors [9,10]. Ferulic acid is an abundant phenolic compound and is found in some major constituents in vegetables and grains, especially in rice bran, nuts, tomatoes, carrots, artichokes, and sweet corn [11]. A previous study showed that rice bran oil, source of ferulic acid, could improve the risk factors for CVD in hyperlipidemic patients [12]. Ferulic acid plays an important role in anti-inflammation by inhibiting cyclooxygenase (COX-2), prostaglandin E2 (PGE2), and tumor necrosis factor—alpha (TNF-α), as evidenced by improved vascular endothelial function both in vivo and in vitro [13,14]. Furthermore, ferulic acid can exert an anti-oxidant effect which is beneficial for cancer prevention by quenching reactive oxygen species (ROS), thereby inducing apoptosis and proliferative reduction [13,15,16]. Moreover, ferulic acid could reduce the level of HMG-CoA reductase, the key enzyme of cholesterol synthesis [14,17,18].

Therefore, ferulic acid might have potential to improve lipid profiles, oxidative stress, and inflammation. It could be a novel alternative nutraceutical for CVD prevention. To date, a clinical study of ferulic acid supplementation in hyperlipidemic patients has not been conducted. The objective of this study is to investigate the effects of ferulic acid supplementation on lipid profiles, oxidative stress, LDL-C oxidation, and inflammatory status in hyperlipidemic subjects. 

## 2. Materials and Methods

### 2.1. Subjects and Study Design

Forty-eight hyperlipidemic subjects, aged 20–60 years, were included, and all had, LDL-C ≥ 130 and ≤190 mg/dL, hs-CRP ≥ 1 mg/L and ≤10 mg/L. The study was conducted at the Faculty of Medicine, Ramathibodi Hospital, Mahidol University, Thailand. Subjects were excluded if they were taking a hypolipidemic drug, had a history of chronic diseases (e.g., diabetes mellitus, hypertension, liver disease, and renal disease), were using herbs or dietary supplements, or smoked or drank alcohol.

The power calculation was based on the ability to detect a 5% difference in LDL-C in the primary analysis of the ferulic acid vs. the control, assuming a 10% SD of effect (α = 0.05 and β−1 = 0.8) and an anticipated drop-out rate of 10%. To satisfy these specifications, 48 subjects were required and were recruited. 

The study was approved by the Ethical Review Committee for Human Research, Faculty of Medicine, Ramathibodi Hospital, Mahidol University (MURA 2009–1666). Furthermore, this study was conducted in accordance with the Declaration of Helsinki on human subjects. All participants were informed and gave their consent before enrollment (clinical trial registration: TCTR20180419005, www.clinicaltrials.in.th).

The study design was a randomized, double-blind, and placebo-controlled trial. Participants were randomly assigned to ferulic acid (Tsuno Food Industrial Co., Ltd., Wakayama, Japan) (treatment group) or maltodextrin as placebo (control group). The random allocation sequence provided by an independent consultant, was computer generated using a randomization plan from www.randomization.com with randomization in blocks of 10. A list of consecutive study numbers was generated. Treatment groups were allocated by research assistant, but the allocation was concealed by assigning each participant with a unique number. Participants, principal investigators, and research assistant were blinded to group allocation. Placebo capsules were manufactured to match the ferulic acid capsules in size, excipients, and appearance. The treatment group were asked to consume a 500 mg capsule of ferulic acid after breakfast and dinner daily (twice daily) for six weeks, during which time the subjects were asked to maintain their habitual diet and lifestyle. All capsules in both groups were similar in shape, size, and color. Anthropometric assessment was conducted by a research assistant. Dietary assessment was collected by food record and calculated by using INMUCAL-nutrient computer program (Institute of Nutrition, Mahidol University, Nakhonpathom, Thailand). The primary outcomes, lipid profiles (total cholesterol, TC; triglyceride, TG; low-density lipoprotein-cholesterol, LDL-C; high-density lipoprotein-cholesterol, HDL-C) and biomarkers of oxidative stress (biological antioxidant potential, BAP; derivatives of reactive oxygen metabolites, dROMs; malondialdehyde, MDA; LDL-C oxidation), and secondary outcomes, inflammatory markers (high sensitivity-C reactive protein, hs-CRP; tumor necrosis factor alpha, TNF-α; interleukin-6, IL-6), were assessed before and after the intervention. 

### 2.2. Biochemical Analysis

Blood samples were collected by a registered nurse after an overnight fast at baseline and at the end of the intervention (six weeks) for biochemical assessments. Fasting blood glucose was determined by the glucose oxidase method [19]. Lipid profiles were determined by an enzymatically colorimetric assay [20]. BAP was measured using an automatic chemistry analyzer [21]. dROMs were determined by detecting hydrogen peroxide spectrophotometrically [22]. MDA was measured by the thiobarbituric acid reactive substances (TBARS) assay [23]. Oxidized LDL-C was determined by enzyme-linked immunosorbent (ELISA) assay [24]. hs-CRP was determined by the latex immunoturbidimetry assay [25]. TNF-α and IL-6 were measured by ELISA assays [26]. All biochemical analyses were carried out at N Health Asia Lab, Bangkok, Thailand, which is a medical laboratory with ISO15189:2007 certification. 

### 2.3. Statistical Analysis

General characteristics and demographic data are presented as mean ± standard deviations (SD). An independent *t*-test was used to analyze the differences in weight, body mass index (BMI), waist circumference, body fat, blood pressure, fasting blood glucose, lipid profiles, oxidative stress markers, and inflammatory markers between the intervention group and control group. A paired *t*-test was used to analyze laboratory results within group. All statistical data were analyzed by using SPSS version 18 (IBM, New York, NY, USA) for Windows. Statistical significance was considered as *p*-value < 0.05.

## 3. Results

Forty-eight hyperlipidemic subjects were recruited and enrolled in this study. General characteristics are presented in Table 1. Age, weight, BMI, waist circumference, percent body fat, blood pressure, blood pressure, fasting blood glucose, liver function, and kidney function test results were not significantly different between two groups before and after the intervention. There were also no significant differences in mean daily energy and nutrient intakes between the two groups before and after the intervention (Table 2). No subjects reported any adverse effects resulting from the consumption of either the ferulic acid or placebo capsules throughout the whole trial period.

All subjects randomly assigned to the two intervention groups completed the study. According to the count of the recalled capsules at the end visit, compliance was very good. The rates of capsule intake were 97% and 98% in the placebo and ferulic acid groups, respectively. 

Lipid profiles, oxidative stress, and inflammatory markers at baseline and at six weeks are presented in Table 3. The baseline concentrations of these lipids, oxidative stress and inflammatory biomarkers did not differ significantly between the two groups. After the six week intervention, ferulic acid supplementation had a significant lowering effect on lipid profiles by the decreasing TC (8.07%; *p* = 0.001*)*, TG (12.12%; *p* = 0.049), and LDL-C (9.32%; *p* < 0.001). Moreover, ferulic acid consumption significantly increased HDL-C (4.32%; *p =* 0.045) compared with the placebo. 

Ferulic acid supplementation significantly decreased the levels of oxidative stress markers, d-ROMs (11.72%; *p* < 0.001), MDA (24.46%; *p* < 0.001), and oxidized LDL-C (7.05%; *p =* 0.002), whereas BAP was increased significantly (11.83%; *p* < 0.001) compared with the placebo. Moreover, the ferulic acid group demonstrated a statistically significant reduction of inflammatory markers, hs-CRP (32.66%; *p* < 0.001) and TNF-α (13.06%; *p* < 0.001) compared with the placebo group, however, IL-6 was not detectable in either group.

## 4. Discussion

Here, we showed for the first time in a clinical study that ferulic acid supplementation improves lipid profiles, oxidative stress, and inflammatory status in hyperlipidemic subjects. The results showed that the six week intervention did not affect anthropometric components such as weight, BMI, waist circumference, body fat, and biochemical markers related to kidney and liver function (Table 1). Thus, a supplement containing 500 mg of ferulic acid twice a day over a short-term period had no effects on body fat and body weight and also no adverse effects on kidney and liver function. 

Our data demonstrated that ferulic acid supplementation can decrease levels of TC, LDL-C, and TG. Similarly, previous studies have reported that ferulic acid improves lipid levels in vitro; and the outcome of animal studies indicate that the probable mechanism of action is via inhibition of HMG-Co A reductase, which controls cholesterol synthesis and modulates the expression of lipogenic genes in the liver [14,17,18,27]. The mechanism by which ferulic acid increases HDL-C levels remains unclear. However, exercise behavior was not the inducible factor in our study due to a non-significant difference in exercise pattern between the treatment and placebo group. The effect of ferulic acid on HDL-C may be similar to that exhibited by the phenolic compounds in cacao, especially flavonoid, which elevated HDL-C [28]. Moreover, dark chocolate and red wine, which are rich in phenolic compounds, affected macrophage cholesterol efflux, which is strongly associated with the ability of Apolipoprotein A-1 (ApoA-1), lecithin activation by cholesterol acyltransferase (LCAT) and mature HDL-C formation [29,30,31]. Evidence from a meta-analysis, 19 randomized controlled trials (RCTs) showed that cocoa flavanol intake significantly improved lipid profile. The amount of cocoa flavanols ranged from 166 to 2110 mg/day, and intervention duration ranged from two to 52 weeks [32].

Ferulic acid supplementation had a positive effect on BAP, which showed the potential effect of its anti-oxidant activity, and is consistent with decreased d-ROMs, MDA, and oxidized LDL-C. This finding was in accordance with a previous study that demonstrated the anti-oxidant effect of ferulic acid in rats [33]. Furthermore, in mice fed western diet, ferulic acid had a beneficial effect on foam cell related atherosclerotic plaques because of its anti-oxidant and anti-lipid peroxidation properties. These properties included activation of anti-oxidant enzymes such as superoxide dismutase (SOD), catalase (CAT), and glutathione peroxidase (GSH) [34]. Regarding anti-inflammatory effects, ferulic acid supplementation could reduce the levels of hs-CRP and TNF-α significantly. These data supported the results from previous studies showed that ferulic acid inhibits oxidative stress and inflammation in rat vascular smooth muscle cells via inhibition of the NADPH oxidase and NF-κB pathway [35]. Additionally, recent research carried out in animal models indicates that ferulic acid decreases TNF-α expression by inhibiting NF-κB signaling [36]. In addition, a pilot human study (five volunteers) presented that the consumption of 500 mg ferulic acid per day for 15 days can reduce oxidative stress human mononuclear cells [37].

The result of ferulic acid supplementation in this study is similar to that of ferulic acid rich foods consumption. Ferulic acid-enriched rice bran oil consumption can decrease LDL-C levels in hyperlipidemic patients [12]. Furthermore, a recent clinical trial showed that supplementation with rice bran extract can reduce LDL-C and TNF-α levels in post-menopausal women with high LDL-C levels [38]. Previous study reported that rice bran consists of ferulic acid about 9000 mg/kg [39]. Thus, the intake of 1,100 g rice bran will be obtainable from the amounts we tested. Additionally, nuts, especially walnuts, rich in phenolic compounds including ferulic acid can improve lipid profile, increase anti-oxidant status, and decrease inflammation in human studies [40].

Recently, the kinetics of ferulic acid and ferulic acid derived metabolites absorption were studied in human plasma before and after acute consumption of 500 mg ferulic acid in five healthy volunteers. Twenty-one phenolic metabolites were identified. The most abundant metabolites were ferulic acid, ferulic acid sulfate, ferulic acid glucuronide, hydroxyphenylacetic acid sulfate, and dihydroxyphenylacetic acid. Ferulic acid, ferulic acid sulfate, and ferulic acid glucuronide showed typical first-order absorption curves, with one absorption peak between 1 and 3 h followed by a clearance period which nearly reached the baseline after 24 h. However, hydroxyphenylacetic acid sulfate and dihydroxyphenylacetic acid plasma concentrations increased with time over the 24-h period, reaching a plateau after 6 h. When the areas under the curve (AUCs) of the absorption curves were calculated, 18.3 ± 6.0 mg free and nonmetabolized ferulic acid and 493.5 ± 27.2 mg of FA equivalents derived from metabolites were found [37]. 

Based on our result and previously published data, we propose the following scenario by which ferulic acid supplementation improve lipid profile, oxidative stress, and inflammation markers. Ferulic acid can inhibit HMG-Co A reductase resulting in TC and LDL-C reduction. Moreover, the anti-oxidant effect of ferulic acid may decrease lipid peroxidation causing decreases of MDA levels and oxidized LDL-C. Also, ferulic acid may inhibit NF-κB, a transcription factor controlling inflammatory gene expression, resulting in decrease of TNF-α and hs-CRP levels.

A limitation of our study was that ferulic acid metabolites were not quantified in plasma. Future study, ferulic acid and its metabolites in plasma should be detected. Moreover, a long-term study of ferulic acid supplementation is needed to confirm the efficacy and safety. 

## 5. Conclusions

Taken together, these data demonstrated that ferulic acid supplementation can improve lipid profiles, oxidative stress and inflammation in hyperlipidemic subjects. These data suggested that ferulic acid supplementation may lower the risk of cardiovascular disease. Therefore, ferulic acid may be an alternative medicine for hyperlipidemia.

## Figures and Tables

**Table 1:** General Characteristics and Blood Chemistry of Subjects.

Parameters	Placebo		Ferulic Acid		P1	P2
	Baseline	6 Week	Baseline	6 Week		
Age (years)	45.88 ± 7.84	-	48.71 ± 7.55	-	0.209	
Sex (*n*)						
-Male	3	3	3	3		
-Female	21	21	21	21		
Weight (kg)	65.32 ± 19.25	65.24 ± 19.53	64.37 ± 15.87	64.34 ± 16.06	0.852	0.863
Body Mass Index (kg/m^2^)	26.58 ± 6.20	26.54 ± 6.29	25.88 ± 5.31	25.86 ± 5.36	0.676	0.689
Waist circumference (cm)	88.80 ± 11.48	88.52 ± 11.43	87.99 ± 10.54	87.72 ± 11.03	0.800	0.805
Body fat (%)	33.84 ± 5.89	33.43 ± 6.31	34.95 ± 3.97	34.32 ± 4.18	0.445	0.567
Blood pressure (mm Hg)						
-Systolic	123.04 ± 19.06	117.83 ± 14.25	123.17 ± 17.3	123.08 ± 15.36	0.981	0.226
-Diastolic	80.38 ± 11.29	77.08 ± 12.53	80.29 ± 10.16	77.04 ± 10.09	0.979	0.990
Glucose (mg/dL)	88.58 ± 7.76	85.63 ± 7.73	84.96 ± 8.01	82.79 ± 8.72	0.118	0.240
ALT (U/L)	18.67 ± 4.80	18.08 ± 4.47	18.42 ± 5.39	18.67 ± 5.50	0.866	0.689
AST (U/L)	22.00 ± 6.65	21.67 ± 6.81	21.46 ± 5.29	20.17 ± 4.99	0.756	0.389
Creatinine (mg/dL)	0.77 ± 0.11	0.78 ± 0.12	0.75 ± 0.13	0.74 ± 0.13	0.491	0.330

Values are means ± SD. P1 = Comparison of mean between the two groups at baseline; P2 = Comparison of mean between the two groups at six weeks; Significant differences at *p* < 0.05. ALT, alanine aminotransferase; AST, aspartate transaminase.

**Table 2:** Total Energy and Nutrient Intake of Subjects.

Dietary Assessment	Placebo		Ferulic Acid		P1	P2
	Baseline	6 Week	Baseline	6 Week		
Energy (kcal/day)	1914 ± 476	1878 ± 399	1854 ± 519	1866 ± 503	0.704	0.931
Carbohydrate (% of energy)	56.74 ± 9.53	55.87 ± 7.67	55.41 ± 9.71	54.77 ± 7.90	0.665	0.659
Protein (% of energy)	15.17 ± 4.79	14.61 ± 4.53	16.37 ± 5.20	16.50 ± 5.11	0.455	0.223
Fat (% of energy)	28.09 ± 6.82	29.52 ± 5.62	28.22 ± 6.70	28.73 ± 4.79	0.949	0.639
Cholesterol (mg/day)	335.60 ± 126.03	351.47 ± 134.98	356.98 ± 110.60	330.54 ± 97.86	0.564	0.572
Fiber (g/day)	10.44 ± 6.14	10.17 ± 6.74	9.27 ± 6.29	10.22 ± 7.99	0.558	0.981

Values are means ± SD. P1 = Comparison of mean between the two groups at baseline; P2 = Comparison of mean between the two groups at 6 weeks; Significant differences at *p* < 0.05.

**Table 3:** Lipid Profile, Oxidative Stress, and Inflammatory Markers.

Biomarkers	Placebo			Ferulic Acid			*p* ^b^
	Baseline ^a^	6 Week	Change (%)	Baseline ^a^	6 Week	Change (%)	
**Lipid profiles**							
TC (mg/dL)	250.83 ± 32.16	245 ± 34.4	−2.17 ± 6.71	254.35 ± 33.65	233 ± 26.26	−8.07 ± 4.56	0.001
TG (mg/dL)	131.5 ± 72.04	122.04 ± 56.47	−0.41 ± 27.85	136.96 ± 59.45	120.22 ± 54.02	−12.12 ± 7.95	0.049
LDL-C (mg/dL)	167.17 ± 27.61	164 ± 28.72	−1.69 ± 6.72	172.74 ± 30.17	155.91 ± 23.73	−9.32 ± 4.82	<0.001
HDL-C (mg/dL)	55.58 ± 13.07	54.71 ± 12.76	−0.94 ± 9.13	51.52 ± 8.82	53.39 ± 7.95	4.32 ± 8.96	0.045
**Oxidative stress markers**							
BAP (μmol/L)	2896.33 ± 256.25	2802.21 ± 222.57	−2.75 ± 9.32	2930.36 ± 345.14	3266.95 ± 386.57	11.83 ± 9.5	<0.001
d-ROMs (CARR U)	323.25 ± 47.9	332.88 ± 56.13	2.98 ± 8.97	358.32 ± 75.91	315.45 ± 63.98	−11.72 ± 5.48	<0.001
MDA (nmol/L)	1092.29 ± 228.41	1015.67 ± 201.56	−6.62 ± 7.3	1155.5 ± 229.52	862.45 ± 166.96	−24.46 ± 10.8	<0.001
Oxidized LDL-C (U/L)	57.48 ± 5.43	56.12 ± 5.47	−2.22 ± 5.93	59.23 ± 3.69	54.98 ± 2.97	−7.05 ± 4.37	0.002
**Inflammatory markers**							
hs-CRP (mg/L)	2.74 ± 1.96	3.27 ± 2.48	25.18 ± 54.89	2.94 ± 1.85	1.82 ± 0.82	−32.66 ± 20.91	<0.001
TNF-α (pg/mL)	42.92 ± 24.62	46.95 ± 24.63	12.75 ± 15.28	44.22 ± 21.14	38.95 ± 20.09	−13.06 ± 6.92	<0.001
IL-6 (pg/mL)	ND	ND	ND	ND	ND	ND	-

Values are means ± SD. ND is non-detectable. ^a^ There were no significant differences between the two groups at baseline; ^b^ Comparison of percentage change between the two groups; significant differences at *p* < 0.05. TC, total cholesterol; TG, triglyceride; LDL-C, low-density lipoprotein-cholesterol; HDL-C, high-density lipoprotein-cholesterol; BAP, biological antioxidant potential; dROMs, derivatives of reactive oxygen metabolites; MDA, malondialdehyde; hs-CRP, high sensitivity-C reactive protein; TNF-α, tumor necrosis factor—alpha; IL-6, interleukin-6.
